# Serum Vitamin D Level and Gut Microbiota in Women

**DOI:** 10.3390/healthcare11030351

**Published:** 2023-01-25

**Authors:** Noorah S. Al-Khaldy, Sara Al-Musharaf, Esra’a A. Aljazairy, Syed Danish Hussain, Abdullah M. Alnaami, Nasser Al-Daghri, Ghadeer Aljuraiban

**Affiliations:** 1 Department of Community Health Sciences, College of Applied Medical Sciences, King Saud University, Riyadh 11451, Saudi Arabia; 2 Biomarkers of Chronic Diseases, Riyadh Biochemistry Department, College of Science, King Saud University, Riyadh 11451, Saudi Arabia

**Keywords:** serum vitamin D, obesity, gut microbiota, microbiome

## Abstract

Obesity and vitamin D deficiency are two major public health concerns. Evidence suggests that alteration in gut microbiota composition is a possible risk factor for obesity. Additionally, altered vitamin D status has a potential role in shaping the gut microbial community. Further, the prevalence of obesity has been rising in the Middle East, especially among women of reproductive age, which is of specific concern due to its adverse effects on the health of their offspring. To date, limited evidence is available on the association between gut microbiota composition and vitamin D levels in Arab women. This study aims to identify the associations between serum vitamin D, gut microbiota, and obesity among Saudi females. The current study is a case–control study including 92 women aged 18 to 25 years, (*n* = 48) with normal weight and (*n* = 44) with obesity. Anthropometric, biochemical, lifestyle data, and fecal samples were collected and analyzed. We used shotgun metagenomic sequencing to characterize microbial communities of stool samples. Vitamin D levels were significantly associated with alpha and beta diversities. Serum vitamin D levels were positively associated with bacteria known to regulate immunological responses; *Bacteroides thetaiotaomicron* in the normal weight group (r = 0.34, *p* = 0.03) and *Bifidobacterium adolescentis* in the obesity group (r = 0.33, *p* = 0.04). In conclusion, the findings suggest that vitamin D status may play a role in regulating the gut microbiota composition by inhibiting the growth of pathogenic bacteria while nourishing the beneficial strains.

## 1. Introduction

Obesity is a complex condition with an increasing prevalence worldwide [1]. Globally, the prevalence of obesity has increased from 7% in 1980 to 12% in 2015 for adult men and women aged 20 years or older, with a higher prevalence in women compared to men (10% vs. 14%) [1]. In Saudi Arabia, the prevalence of obesity and overweight has reached 20% and 38%, respectively, with higher percentages in women compared to men (21% vs. 19%) [2]. In Saudi women of reproductive age, the prevalence of obesity and overweight was 36% and 27%, respectively [3]. These figures are alarming, especially in young women, as the associated complications are life-threatening for both the mother and fetus [4]. Obesity originates due to multiple factors including genetics, unhealthy diet, and sedentary lifestyle [5] along with other recent risk factors such as sleep disturbance [6], vitamin deficiencies [7,8], and gut microbiota imbalance [9,10].

The human gastrointestinal (GI) tract contains a complex population of microorganisms, which creates a diverse microbial community in the gut [11]. Dysbiosis refers to an alteration of microbial composition [12] and has been associated with multiple conditions including obesity [10]. Studies proposed that gut microbiota differs between people with obesity and people with normal weight due to different host gut microbiota homeostasis [9,10]. Additionally, one of the main contributors to microbial imbalance is nutrient level, especially vitamin deficiencies such as vitamin D [9]. Hence, understanding the diversity and composition of the gut microbiota and the factors that influence its imbalance is crucial.

Vitamin D deficiency has increased globally [13], with rates reaching around 30% in the United States in adults aged (20–39 years) during 2007–2010 [14], 40% among European populations aged (15–18 years) in 2016 [15], and 60% among Saudi adults in 2018 [16]. In Saudi Arabia, vitamin D deficiency is more prevalent in women compared to men [17]. Recently, studies have shown that certain sufficient vitamin levels, particularly vitamin D, may modify diversity and the relative abundance of the gut microbiota community, through regulating immunity, maintaining the intestinal barrier, and reducing inflammation in the intestinal environment [18,19,20]. 

Notably, limited studies are available on the association of vitamin D deficiency with the gut microbiota in individuals with obesity. To our knowledge, only two clinical trials [21,22] have interrelated all three factors; vitamin D, gut microbiota, and obesity. 

Saudi Arabia is currently facing fast lifestyle changes and a transition to a Western dietary pattern, characterized by energy-dense, high saturated fat intake [21]. These patterns may lead to micronutrient deficiencies along with dysbiosis, particularly among the young adult population. Globally, health efforts are focused on preventing and managing obesity, highlighting the potential significance of this study in understanding the contribution of the gut microbiome to human health. We aim to identify the relation between serum levels of vitamin D and the gut microbiota among Saudi Arabian females based on adiposity level using the high-standard shotgun technique to identify microbial communities of stool samples.

## 2. Materials and Methods

### 2.1. Study Design, Population, and Sample Size

We analyzed data from a previously published case-control study on gut microbiota and obesity [22]. In the current study, we investigate dietary aspects related to gut microbiota. The study was conducted at King Saud University (KSU) between January 2019 and March 2020. The Institutional Review Board Committee of the Deanship of Scientific Research at KSU (IRB #E-19-3625) approved the study. The participants signed a consent form before their inclusion in the study and had the right to withdraw at any time.

### 2.2. Inclusion and Exclusion Criteria 

The exclusion criteria were as follows: aged ≤ 18 years, pregnancy, GI diseases (e.g., inflammatory bowel disease, history of colon cancer, and chronic or acute diarrhea in the previous eight weeks), history of oncological or endocrine disease, psychiatric disorders, other medical conditions, following specific diets (e.g., calorie-restricted diets), and the use of vitamin supplements or antibiotics during the six months before the stool sampling.

### 2.3. Data Collection 

The anthropometric and biochemical measurements were collected using standardized methods. All used questionnaires were validated and included: a general health history [23], the Saudi Food and Drug Authority-food frequency questionnaire (SFDA-FFQ) [24], a sun exposure questionnaire [25], physical activity [26], and sleep [27].

#### 2.3.1. Anthropometric Assessment 

Trained clinical dietitians performed all measurements using standardized methods. Each measurement was recorded twice, with the mean reading used for the final analysis. The weight of the participants was measured without shoes and with minimal clothes to the nearest 0.1 kg using a proper international scale (Digital Pearson Scale; ADAM Equipment Inc., Oxford, CT, USA). Height was reported while standing and facing the scale to the approximate 0.5 cm without shoes. Body mass index (BMI) was calculated by dividing weight in kilograms by height in meters squared. The participants were classified according to their BMI into: normal weight (18.5–24.9 kg/m^2^) and obese (≥30 kg/m^2^) using the WHO criteria [28].

Hip circumference was measured using non-stretchable tape with legs close together at the point of the great trochanter. Waist circumference was also measured using non-stretchable tape at the narrowest point between the umbilicus and the lowest rib. Both measurements were reported to the nearest 0.5 cm. In addition, the waist–hip ratio (WHR) was calculated by dividing the mean waist circumference by the mean hip circumference [29]. Body fat percentage (BF%) and muscle mass were measured via bioelectrical impedance analysis (770 BIA; InBody, Seoul, South Korea) [30]. 

#### 2.3.2. Biochemical Measurement

Blood samples were collected after an overnight fast (≥10 h) to measure serum levels of vitamin D, lipid profile; (high-density lipoprotein cholesterol (HDL-C), total cholesterol (TC), and TG levels), fasting blood glucose (FBG), and insulin. Blood samples were transported to the study laboratory and were stored immediately at −80°C for further analysis. 

##### Vitamin D

Serum levels of total 25(OH)D were measured using the electrochemiluminescence binding assay (ECLIA). The repeatability and intermediate coefficients of variation (CV) for the 25(OH)D assay were 4.4% and 6.6%, respectively, with 100% cross-reactivity to 25(OH) D3, and 92% cross-reactivity to 25(OH) D2. The measurements ranged between 7.50–175 nmol/L, and any hemolysis samples were excluded. The Biomarker Research Program (BRP) laboratory is a partnership with the vitamin D External Quality Assessment Scheme (DEQAS). Vitamin D status was determined based on 25(OH)D concentrations, as follows:

<25 nmol/L (severe deficiency); 25–49.9 nmol/L (deficiency); 50–74.9 nmol/L (insufficiency), and ≥75 nmol/L (sufficiency) [31,32]. 

##### Stool Sample

Under sterile conditions, fresh stool samples were collected in a clean container and dry screw-top and transported to the study lab, and stored immediately at −80 °C for further analysis.

##### DNA Extraction 

The DNA was extracted from 0.25 frozen stool aliquots using the QIAGEN PowerFecal DNA Kit (Catalogue: 12830-50). The DNA was eluted in 100 microliters of the C6 elution buffer according to the kit’s protocol. Using a NanoDrop spectrophotometer, the isolated DNA purity (260/280a ratio) and concentration (≥1.6) were measured (Thermo Fisher Scientific, Waltham, MA, USA). Until library preparation and sequencing, the extracted DNA samples with a volume of (≥12.5) were stored at (−20 °C) in the CBCD laboratory.

##### Library Preparation and Sequencing

DNA libraries were constructed using the Nextera XT DNA Library Preparation Kit (Illumina, San Diego, CA, USA) and Nextera Index Kit (Illumina) with total DNA input of 1 nanogram. A proportional amount of Illumina Nextera XT fragmentation enzyme was used to fragment genomic DNA. Each sample was provided a combination of dual indexes, followed by 12 cycles of polymerase chain reaction (PCR) to build libraries. AMpure magnetic beads (Beckman Coulter) were used to purify the DNA libraries, which were then eluted in QIAGEN EB buffer. For the quantitative evaluation, a Qubit ^®^ fluorimeter (Thermo Fisher Scientific, Milan, Italy) was used. Following the library preparation, the samples were sequenced on an Illumina HiSeq 4000 (2 × 150 bp).

##### Identification of the Microbial Composition

The gut microbiota composition was determined at the level of the major microbial phyla and was carried out by identifying the total bacterial DNA and the Bacteroidetes and Firmicutes DNA using shotgun metagenomic sequencing that was analyzed using CosmosID bioinformatics.

##### The CosmosID Bioinformatics Platform

For the investigation of the multi-kingdom microbiome and the measurement of gut microbiota relative abundance, we used the CosmosID bioinformatics platform (CosmosID Inc., Rockville, MD, USA). The fine-grain taxonomic and relative abundance estimations for the microbial NGS datasets are obtained after the analysis of the resulting statistics. The findings are filtered using a filtering threshold developed based on internal statistical scores established by examining a large number of varied metagenomes to eliminate false positive identifications. 

##### Relative Abundance Stacked Bars 

Phylum-, genus-, species-, and strain-level filtering matrices for bacteria from CosmosID-HUB were used to create stacked bar graphs. The R package ggpubr was used to create stacked bar graphs for each group [33,34]. The CosmosID-HUB is a database of microbial sequences that can be used to identify and analyze bacteria. The database includes information about the taxonomic classification of each bacterial strain, including its phylum, genus, species, and strain level.

A filtering matrix was used to create stacked bar graphs to select specific groups of bacteria based on their taxonomic classification. For example, the matrix might include information about the phylum, genus, species, and strain level of each bacterial strain and allow the user to select only those strains that belong to a particular phylum, genus, species, or strain.

Once the desired groups of bacteria have been selected using the filtering matrix, the R package ggpubr can create stacked bar graphs that visualize the relative abundance of each group within the dataset. The stacked bar graph will show the relative abundance of each group as a proportion of the total number of bacterial strains in the dataset. This can be useful for understanding the overall composition of a bacterial community and for identifying patterns or trends in the data.

##### Alpha Diversity Boxplots (with Wilcoxon Rank-Sum) 

The phylum-, genus-, species-, and strain-level abundance score matrices from CosmosID-HUB analyses were used to create alpha diversity boxplots. Utilizing the Vegan R package, the Chao, Simpson, and Shannon alpha diversity measures were computed [35,36]. Using the ggsignif R package, Wilcoxon Rank-Sum tests were carried out between groups [37]. Using the R package ggpubr [34], boxplots with superimposed significance and *p*-value formats were created.

##### Beta Diversity PCoA (with PERMANOVA)

Beta Principal Coordinate Diversity phylum-, genus-, species-, and strain-level matrices for bacteria from CosmosID-HUB were used to calculate the analyses. The vegan package’s function vegdist was used to calculate Bray–Curtis dissimilarity in R, and the function PCoA of the ape package was used to create PCoA tables [38]. The vegan function adonis2 [36] was used to create PERMANOVA tests for each distance matrix, and the vegan function betadisper’s ANOVA technique [36] was used to compute and compare beta dispersion. The R package ggpubr was used to show the plots [34].

#### 2.3.3. Questionnaires

##### General Health History Questionnaire 

Data on family medical history, sociodemographic information (family income, marital status, husband’s relativity, college, specialty, university semester level, occupation, living area, and place of residence), and the medical history of the participants were collected [23]. 

##### Dietary Intake 

Macronutrients and micronutrient consumption for the past year were assessed using the SFDA FFQ [24]. The questionnaire was developed in the Arabic language and used a closed-ended approach, including a list of 133 food items. Open-ended questions were included at the end to gather information on other food items that were not listed [24]. The SFDA FFQ was analyzed using the Food Processor Nutrition Analysis Software version 11.1 (ESHA Research, Salem, OR, USA). 

##### Sun Exposure

Information on sun exposure was assessed in seven questions including the current season, the time and duration of sun exposure, sun exposure at work (indoor or outdoor work), clothing (whole-body coverage or some parts of the body exposed), and the use of sunscreen (yes/no) [25].

##### Physical Activity

Using the Arabic version of the GPAQ questionnaire [26], data on the intensity, frequency, and duration of physical activity were collected. Additional data included: transport-related physical activity, occupational, physical activity, and physical activity during discretionary or leisure time. 

##### Sleeping Index: Pittsburgh Sleep Quality Index (PSQI)

Quality of sleep was assessed using the Arabic version of the PSQI questionnaire [27] to evaluate sleep quality during a 1-month period. The following seven components were included: subjective sleep quality, sleep latency, sleep duration, sleep efficiency, sleep disturbance, use of sleeping medication, and daytime dysfunction. A total score of less than 5 indicates good sleep, whereas a score of 5 or higher indicates poor sleep [39].

### 2.4. Statistical Analysis

All statistical analyses were performed using IBM SPSS Statistics for Windows (version 24; IBM Corp., Armonk, NY, USA). The participants were classified based on vitamin D level as follows: severe vitamin D deficiency (<25 nmol/L) and non-severe vitamin D deficiency (≥25 nmol/L); vitamin D deficiency (<50 nmol/L); and non-vitamin D deficiency (≥50 nmol/L). The results are presented according to adiposity level and vitamin D deficiency.

The normality of each quantitative variable was tested before the analysis. Appropriate non-parametric tests were used if the variables showed a skewed pattern. Descriptive analysis results were shown as frequencies and percentages for categorical data characteristics, with means and standard deviations for continuous data characteristics. Associations between categorical variables and the outcomes were identified using Pearson’s chi-squared test, and the independent samples t-test was used for continuous variables and outcomes. The Kruskal–Wallis test (one-way analysis of variance) was used to determine the statistical differences between those with obesity and normal weight and in comparing medians. A *p*-value of <0.05 and a 95% CI were used to report the statistical significance and precision of the estimates. 

## 3. Results

### 3.1. Baseline Characteristics

The average age of the participants was 21.1 ± 1.5 years. Of the 92 Saudi women included in this study, 48% had obesity and 52% had normal weight. The anthropometric and biochemical parameters are presented in Table 1. In the normal weight group, those with severe vitamin D deficiency had significantly higher fat intake (45.7% vs. 36.6%, *p* = 0.029) compared to non-severe vitamin D deficiency. No significant differences in sleep and physical activity were observed between the groups. The lifestyle characteristics among the participants with severe and non-severe vitamin D deficiency relative to body weight are shown in (Supplementary Table S1).

### 3.2. Analyses Performed According to Vitamin D Status and BMI Categories

#### Gut Microbiota Composition

The independent samples *t*-test analysis showed no statistically significant differences in the gut microbiota relative abundances at all taxonomic levels between severe vs. non-severe vitamin D deficiency in those with obesity and those with normal weight (Supplementary Table S2). However, in the normal weight group, there was an increased trend in *Actinobacteria*, Bacteria “unspecified phylum”, *Clostridium bolteae* (*Firmicutes* phylum), *Bacteroides thetaiotaomicron* (*Bacteroidetes* phylum), and *Bifidobacterium pseudocatenulatum* (*Actinobacteria* phylum) species in those with non-severe vitamin D deficiency compared to those with severe vitamin D deficiency (all *p* > 0.05). In the group with obesity, increased *Bifidobacterium adolescentis* species were also observed in those with non-severe vitamin D deficiency in comparison to those with severe vitamin D deficiency, although it was not statistically significant (*p* = 0.22).

### 3.3. The Correlation between BMI, Gut Microbiota, and Vitamin D Status

**Figure 1:**
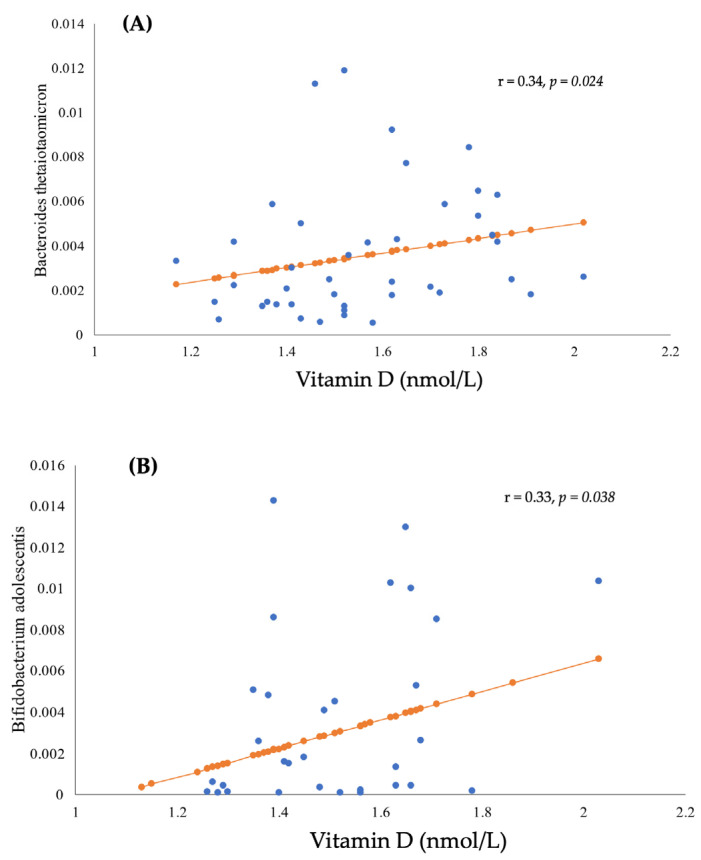
Associations between serum vitamin D and gut microbiota relative abundance (**A**) Association between serum vitamin D and *Bacteroides thetaiotaomicron* species in participants with normal weight (controls). (**B**) Association between serum vitamin D and *Bifidobacterium adolescentis* species in participants with obesity (cases).

Pearson’s correlation showed no association between serum vitamin D levels with gut microbiota relative abundance in the overall sample. In the normal weight group, Pearson’s correlation showed a significant positive correlation between serum vitamin D and *Bacteroides thetaiotaomicron* species (r = 0.34, *p =* 0.02) (Figure 1A). In the group with obesity, a positive correlation was found between *Bifidobacterium adolescentis* species and serum vitamin D (r = 0.33, *p =* 0.04) (Figure 1B).

For vitamin D intake, there was an inverse correlation between vitamin D intake and *Bifidobacterium kashiwanohense* species (r = −0.22, *p* = 0.04) and between vitamin D intake and *Blautia wexlerae* species (r = −0.21, *p* = 0.05) in the total participants (Supplementary Table S3).

In the normal weight group, there was a positive correlation between vitamin D intake and *Akkermansia muciniphila* species (r = 0.29, *p* = 0.03) and a negative correlation with *Flavonifractor plautii* species (r = −0.33, *p* = 0.02).

In the obese group, a positive correlation was found between *Bacteroides* “unspecified species” and vitamin D intake (r = 0.35, *p* = 0.02). Furthermore, *Blautia wexlerae* species (r = −0.30, *p* = 0.05), *Akkermansia muciniphila* species (r = −0.32, *p* = 0.04), and *Actinobacteria* phylum (r = −0.35, *p* = 0.02) were inversely correlated with vitamin D intake (Supplementary Table S3)

### 3.4. Gut Microbiota Analyses and Serum Vitamin D 

#### 3.4.1. Relative Abundance 

**Figure 2:**
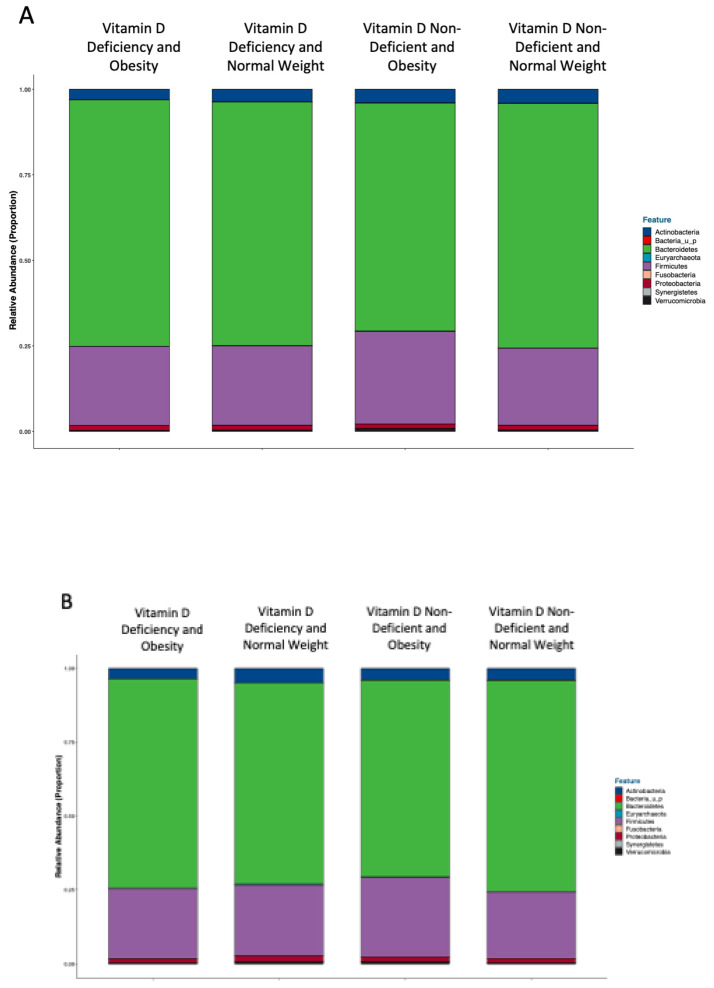
Classification of gut microbiota at the phylum level according to categories of vitamin D deficiency. (**A**) Classification of gut microbiota at the phylum level according to categories of vitamin D deficiency (<50 nmol/L) relative to obesity (<50 nmol/L) and relative to BMI. (**B**). Classification of gut microbiota at the phylum level according to categories of severe vitamin D deficiency (<25 nmol/L) relative to obesity.

Different cutoffs (<25 nmol/L) and (< 50 nmol/L) were applied to have a better understanding of gut microbes with different vitamin D serum levels. The most abundant phyla in those with vitamin D deficiency and obesity, using the cut-off (<50 nmol/L), were *Bacteroidetes* (71.45%), *Firmicutes* (23.37%), and *Actinobacteria* (3.37%) (Figure 2A). Using the cut-off (<25 nmol/L), the most abundant phyla among obese participants with severe vitamin D deficiency were *Bacteroidetes* (71.65%), *Firmicutes* (23.37%), and *Actinobacteria* (3.22%) (Figure 2B).

#### 3.4.2. Diversity

**Figure 3:**
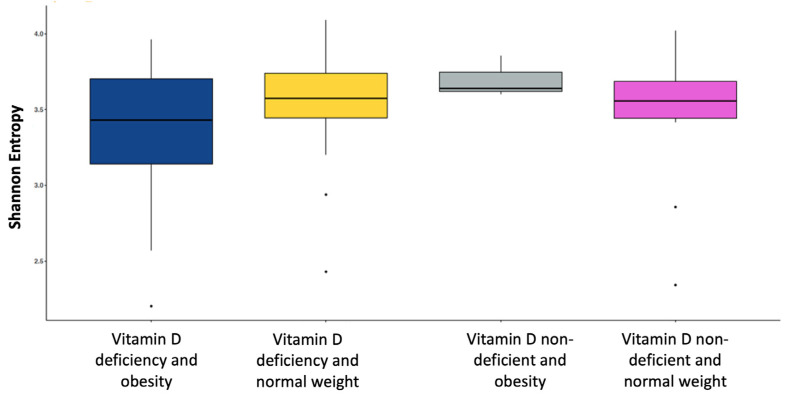
Comparison of bacterial diversity at the species level (Shannon-Wiener index) between participants with obesity and participants with normal weight based on vitamin D level (vitamin D deficiency < 50 nmol/L).

**Figure 4:**
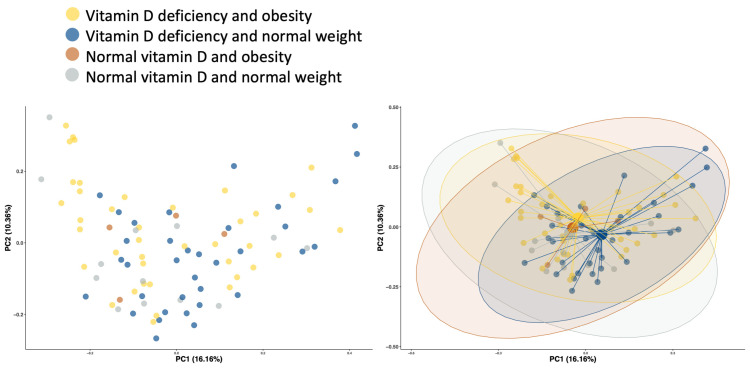
PCoA plot of the Bray–Curtis dissimilarity index.

For severe vitamin D deficiency (<25 nmol/L), there were no significant results in alpha or beta diversity (Supplementary Figures S1 and S2). Using the vitamin D deficiency cut-off (<50 nmol/L), the bacterial diversity by obesity level is shown in Figure 3 and Figure 4. The Shannon–Wiener index showed a significant difference in the alpha diversity between those with vitamin D deficiency (<50 nmol/L), in the obesity and normal weight groups. The participants with vitamin D deficiency in the obesity group had lower alpha diversity than the normal weight group (median: 4.9 vs. 5.2; *p* = 0.03, Figure 3). 

To further identify whether the structure of the bacteria in gut microbiota is similar between the vitamin D-deficient groups (participants with obesity vs. with normal weight), the principal coordinate analysis (PCoA) plots based on the Bray–Curtis dissimilarity Index were analyzed. We observed a significant difference in the beta diversity between the vitamin D-deficient groups by PCoA; the results were showed by principal coordinates (PCs): PC1 and PC2 accounted for 16.16% and 10.36% (Bray–Curtis index, *p* = 0.017, Figure 4). 

## 4. Discussion

In this study, we found that serum vitamin D deficiency and obesity were inversely associated with the alpha diversity of gut microbiota. We also found that serum vitamin D levels were positively associated with *Bacteroides thetaiotaomicron* in the normal weight group, and with *Bifidobacterium adolescentis* in those with obesity. 

In the last decade, the number of studies on serum vitamin D and gut microbiota has increased; the majority have been clinical trials [40,41,42,43,44,45,46,47,48], and a few have been observational studies [45,49,50,51]. Some observational studies have shown that vitamin D concentration is associated with an abundance of specified gut bacterial composition [43,45,49,50]. A cross-sectional study (nested with a randomized controlled trial) identified an association between high serum vitamin D levels and gut microbiota composition in 115 prediabetic patients with vitamin D deficiency [43]. The results showed a significant decrease in Firmicutes (*Ruminococcus, Dorea, Blautia*, and *Roseburia*) with increased serum vitamin D levels [43]. The mechanism could be explained by the role of vitamin D and its receptor in gut microbiota composition and microbiota-induced inflammation, which is supported by in vitro and in vivo studies [52]. Vitamin D receptors (VDR) and enzymes are available in colon epithelial cells and are involved in the metabolization of active vitamin D (1,25(OH)_2_D_3_) [53,54]. The mechanisms of vitamin D actions are mostly indirect, as bacteria do not express the VDR and include the maintenance of the intestinal barrier, the activation of innate immunity, the generation of antimicrobial peptides, and changes in calcium and phosphorous absorption [18]. Vitamin D regulates innate immunity in multiple ways, one of which is the upregulation of a receptor known as nucleotide-binding oligomerization domain-containing protein 2 (NOD2) in human monocytes, which results in bacterial killing [55]. Dysregulation in NOD2 leads to reduced antimicrobial peptide expression, impaired autophagy, and dysbiosis [55]. In particular, active vitamin D stimulates macrophages to produce antimicrobial peptides, such as beta-defensin and cathelicidin [18].

It is worth noting here that studies investigating the association between vitamin D, gut microbiota and obesity are limited, only two studies have examined all three variables together [46,56] identifying different bacterial associations than the ones we presented in our study. The Naderpoor et al. trial found a higher composition of the genus *Coprococcus* and a lower composition of the genus *Ruminococcus* (both of them from the phylum *Firmicutes*) in those with higher vitamin D levels [46]. Barrea et al. showed that high levels of TMAO (a gut microbiota-generated metabolite) were associated with vitamin D deficiency (<50 nmol/L) and linked with the severity of obesity [56].

In the normal weight group, serum vitamin D was positively correlated with the *Bacteroides thetaiotaomicron* species, which are anerobic Gram-negative bacteria [57,58] that regulate the host’s immunological responses and stimulate antimicrobial protein production [58,59], indicating its role in maintaining a healthy gut. No previous studies have investigated the relation between *Bacteroides thetaiotaomicron* and serum vitamin D. However, a study on genome-scale models of gut bacteria by Gokhale and Bhaduri showed that prebiotic supplementation can help enhance the biosynthesis of provitamin D induced by the human *Bacteroides thetaiotaomicron* [60]. It is interesting to note that the reduced composition of *Bacteroides thetaiotaomicron* was reported in patients with inflammatory bowel diseases [58]. Vitamin D plays a role in several physiological mechanisms, including immunity [61], as does *Bacteroides thetaiotaomicron* in regulating immunological responses [58]. 

In the group with obesity, serum vitamin D was positively correlated with the *Bifidobacterium adolescentis* species, one of the most predominant bacteria in the gut of healthy adults [62,63]. *Bifidobacterium adolescentis* can help the host against gut infection through the stimulation of macrophages and the inhibition of pathogens [64]. Up to date, no studies were found on the association between *Bifidobacterium adolescentis* and the serum level of vitamin D. Our findings indicate that as vitamin D plays a role in inflammatory diseases [65], *Bifidobacterium adolescentis* species may inhibit inflammation through immunoregulatory properties [63,66]. In addition, *Bifidobacterium* species showed an inverse association with body weight in an experimental study of rats [66]. The supplementation of *Bifidobacterium adolescentis* also improved visceral fat accumulation and insulin sensitivity in rats fed a high-fat diet [67].

In the total sample and in those with obesity, the *Blautia wexlerae* species was inversely correlated with vitamin D intake, indicating that the composition of the *Blautia wexlerae* species may be decreased with adequate vitamin D intake. This finding supports previous results that vitamin D may change the gut microbiota by reducing *Firmicutes* and increasing *Bacteroidetes* [68]. Yet, the composition of *Blautia wexlerae* species in the gut may also be decreased with obesity, leading to gut impairment and inflammation [69]. 

No studies up to date have reported the association between the alpha and beta diversity of the gut microbiota and vitamin D relative to obesity. Alpha diversity is used to assess the evenness and richness of the gut microbiota, whereas beta diversity is used to evaluate its composition for comparison between the two communities [70]. The quality of human health has been linked to gut microbiome alpha diversity, and lower levels of alpha diversity are related to many acute and chronic diseases [71,72]. We found that vitamin D deficiency was inversely associated with the alpha diversity of the gut microbiota relative to obesity. The participants with vitamin D deficiency and obesity had lower alpha diversity than the normal-weight participants with vitamin D deficiency (median: 4.9 vs. 5.2; *p* = 0.03). It is well known that vitamin D deficiency is associated with obesity [73]; however, limited studies have measured alpha diversity and obesity [74,75], and fewer studies have assessed alpha diversity with vitamin D [46,76]. Low alpha diversity may negatively aggravate health status. Studies suggest that a reduction in microbiota richness and evenness (alpha diversity) is an indication of dysbiosis and associated diseases [77,78]. This association indicates the negative impact of vitamin D deficiency on health status. The trial by Naderpoor et al. showed no significant differences in alpha diversity between vitamin D-supplemented and placebo groups; however, a reduction in alpha diversity was observed among vitamin D-supplemented groups at follow-up in comparison with baseline [46]. In addition, a cohort study using data from the American Gut Project assessed the relationship between a history of vitamin B and/or D supplementation and gut microbiota in groups of people with overweight or normal weight. The results showed no association between microbial alpha diversity and vitamin D supplementation among normal-weight and overweight populations [76]. The different techniques used for the gut microbiota sequencing between our study and the trial and cohort studies may contribute to inconsistent findings. Moreover, most of the participants in the cohort study were Caucasian [76]. Recent evidence revealed that alpha diversity was strongly associated with ethnicity, highlighting the role of ethnicity in the interindividual differences of gut microbiota [79].

Our study found a significant difference in beta diversity between normal-weight and group with obesity based on vitamin D deficiency, with PC1 and PC2 accounting for 16.16% and 10.36% (*p* = 0.017). Similarly, a recent systematic review of human studies on the association between vitamin D and gut microbiota revealed that vitamin D supplementation and serum 25(OH)D levels are associated with significant changes in bacterial community composition [80], emphasizing the variation of microbial communities between the study cohorts. In contrast, an interventional, open-label pilot study of 16 healthy adults showed that vitamin D supplementation significantly changed beta diversity in upper GI biopsies, but not in feces or lower gastrointestinal biopsies [40]. The variability in obtaining the sample from different anatomical parts of the GI tract or the technique used may lead to inconsistent results. Thus, the need for future studies addressing alpha and beta diversity, vitamins, and body weight is crucial to give insight into these associations.

The current study is the first to investigate the association between serum levels of vitamin D and gut microbiota relative to obesity. We used extensive measures in data collection to improve accuracy and reduce errors. To reduce confounding effects, we excluded participants under 18 years old, those following specific diets, those using vitamin supplements or antibiotics, and those with GI diseases or other medical conditions. The study used the whole-genome shotgun sequencing method in taxonomic identification to species level [81]. Nonetheless, several limitations are presented in this study, i.e., as the present study analyzed data from a previously published case-control study on gut microbiota and obesity, its design prohibits causal inference between vitamin D status and gut microbiota among women in Saudi Arabia relative to obesity status. Moreover, this study only involved females; therefore, caution should be exercised when generalizing its findings as they might not represent the whole Saudi population. Although we used validated questionnaires such as FFQ, GPAQ, and PSQI, information errors caused by recall bias may affect the interpretation of the results. 

## 5. Conclusions

This study aimed to explore the association between vitamin D status and gut microbiota among women in Saudi Arabia relative to obesity status. We found that vitamin D deficiency was inversely associated with alpha diversity. Additionally, we observed a significant difference in beta diversity between the vitamin D-deficient groups. Further, serum vitamin D levels were associated with bacteria known to regulate immunological responses, such as *Bacteroides thetaiotaomicron* in the normal weight group, and *Bifidobacterium adolescentis* in the obesity group. These associations provide insight into the possible benefits of sufficient vitamin levels for health through gut microbiota. Our findings highlight the role of vitamin D status in gut microbiota and obesity and warrant further experiments that focus on understanding the mechanisms of the bacteria identified in this study. Additionally, considering the genetic profile in studies investigating the three variables (vitamin D, obesity, and gut microbiota) is necessary to achieve individualized plans in clinical applications.

## Figures and Tables

**Table 1:** Anthropometric and biochemical parameters of participants according to body mass index and vitamin D status ^1^.

Variables	Non-Obese			Obese		
	Vitamin D > 25 nmol/L	Vitamin D < 25 nmol/L	*p*-Value	Vitamin D > 25 nmol/L	Vitamin D < 25 nmol/L	*p*-Value
N	*n* = 34	*n* = 9		*n* = 24	*n* = 16	
Age (y)	20.6 ± 1.1	20.3 ± 1.1	0.550	21.5 ± 1.8	21.5 ± 1.7	1.000
Anthropometric measurements						
BMI (kg/m^2^)	54.1 ± 6.6	53.9 ± 5.2	0.917	89.5 ± 12.3	89.2 ± 12.5	0.947
Waist circumference (cm)	21.7 ± 1.9	22.0 ± 2.0	0.661	35.8 ± 4.8	36.6 ± 5.1	0.609
Hip circumference (cm)	68.1 ± 4.0	66.2 ± 3.7	0.220	95.4 ± 10.2	96.3 ± 18.1	0.840
WHR (ratio)	96.5 ± 8.3	94.4 ± 6.1	0.476	123.9 ± 9.9	123.7 ± 13.2	0.949
Body fat (%)	0.7 ± 0.1	0.7 ± 0.0	0.787	0.8 ± 0.1	0.8 ± 0.1	0.549
Blood parameters						
Vitamin D (nmol/L)	**39.6 (31.9–59.7)**	**19.7 (18.0–22.8)**	**<0.001**	**40.0 (32.7–46.4)**	**19.8 (18.6–23.8)**	**<0.001**
Total cholesterol (mmol/L)	**4.0 ± 1.7**	**2.8 ± 1.4**	**0.057**	**4.5 ± 1.0**	**4.5 ± 1.0**	**0.899**
HDL cholesterol (mmol/L)	**1.0 ± 0.4**	**0.9 ± 0.4**	**0.760**	**1.0 ± 0.3**	**1.0 ± 0.3**	**0.814**
LDL cholesterol (mmol/L)	**2.9 ± 1.5**	**1.8 ± 1.0**	**0.048**	**3.3 ± 1.0**	**3.4 ± 1.0**	**0.877**
Total cholesterol/HDL ratio	**4.2 ± 1.8**	**3.0 ± 0.6**	**0.065**	**4.7 ± 1.8**	**4.6 ± 1.5**	**0.845**
Triglyceride (mmol/L) #	**0.6 (0.4–0.8)**	**0.4 (0.3–0.5)**	**0.034**	**1.0 (0.9–1.2)**	**0.9 (0.7–1.1)**	**0.266**
FBG (mmol/L)	**4.5 ± 0.7**	**4.6 ± 1.0**	**0.702**	**4.9 ± 0.6**	**4.6 ± 0.6**	**0.168**
Insulin (µIU/mL) #	**5.8 (4.6–9.2)**	**10.9 (6.9–15.3)**	**0.009**	**14.9 (12.7–18.3)**	**16.0 (10.7–20.4)**	**0.619**
HOMA-IR #	**1.1 (0.9–1.5)**	**1.6 (1.2–3.0)**	**0.042**	**3.5 (2.5–4.0)**	**3.6 (2.2–4.8)**	**0.885**
HOMA-β #	106.6 (65.4–142.9)	131.3 (92.5–246.9)	0.161	215.2 (165.7–319.2)	274.8 (192.5–395.4)	0.364

^1^ Data presented as mean ± SD for normal variables, whereas median (1st quartile–3rd quartile) for non-normal variables; # indicates non-normal variables; *p* < 0.05 considered significant. Body mass index (BMI), waist-to-hip ratio (WHR), high-density lipoprotein (HDL), low-density lipoprotein (LDL), fasting blood glucose (FBG), homeostatic model assessment of insulin resistance (HOMA-IR), and homeostatic model assessment of β-cell function (HOMA-β).

## Data Availability

Not applicable.

## Supplementary Materials

The following supporting information can be downloaded at: https://www.mdpi.com/article/10.3390/healthcare11030351/s1, Table S1. General Characteristics of the Subjects with Severe Vitamin D Deficiency in Comparison to Not Severe- Gut Microbiota Composition. Table S2. Lifestyle Parameters of Participants According to Body Mass Index and Vitamin D status. Table S3. Correlation coefficients between vitamin D intake and gut microbiota among study groups. Figure S1. Comparison of bacterial diversity (Shannon-Wiener index) between the microbiota at the species level of group with obesity and group with normal weight based on BMI and severe vitamin D deficiency (<25 nmol/L). Figure S2. Comparison of bacterial diversity (PERMANOVA Analysis) between the microbiota at the species level of normal weight and participants with obesity based on BMI and severe vitamin D deficiency (<25 nmol/L).
