# Supplementary material for: Serum Vitamin D Level and Gut Microbiota in Women
Source: Healthcare (Basel). 2023 Jan 25;11(3):351. doi: 10.3390/healthcare11030351 (PMC9914434; doi:10.3390/healthcare11030351)
Supplement: Supplementary file 1 [file healthcare-11-00351-s001.zip › healthcare-2092688-supplementary.pdf]

Supplementary Table S1. General Characteristics of the Subjects with Severe Vitamin D Deficiency in Comparison to Not Severe- Gut Microbiota Composition

Variables	Severe Vitamin D Deficiency		*p*-value
	**Vitamin D ≥ 25 nmol/l**	**Vitamin D < 25 nmol/l**	
	**n=67**	**n=25**	
**Firmicutes**	0.2177 (0.1598 - 0.2975)	0.1945 (0.1652 - 0.2603)	0.572
Blautia wexlerae	0.0060 (0.0030 - 0.0089)	0.0048 (0.0030 - 0.0109)	0.929
Flavonifractor plautii	0.0006 (0.0003 - 0.0012)	0.0008 (0.0004 - 0.0015)	0.736
Clostridium bolteae	0.0004 (0.0000 - 0.0008)	0.0003 (0.0000 - 0.0005)	0.422
Faecalibacterium prausnitzii	0.0185 (0.0129 - 0.0274)	0.0216 (0.0121 - 0.0320)	0.512
Lactobacillus acidophilus	0.0000 (0.0000 - 0.0000)	0.0000 (0.0000 - 0.0000)	0.789
Clostridioides difficile	0.0000 (0.0000 - 0.0000)	0.0000 (0.0000 - 0.0000)	0.211
**Bacteroidetes**	0.7181 (0.6281 - 0.7874)	0.7522 (0.7079 - 0.7868)	0.341
Bacteroides "unspecified species"	0.0016 (0.0004 - 0.0047)	0.0028 (0.0009 - 0.0049)	0.356
Bacteroides faecichinchillae	0.0000 (0.0000 - 0.0000)	0.0000 (0.0000 - 0.0000)	0.926
Bacteroides thetaiotaomicron	0.0057 (0.0032 - 0.0124)	0.0051 (0.0032 - 0.0105)	0.770
Bacteroides uniformis	0.0629 (0.0327 - 0.0841)	0.0753 (0.0532 - 0.1140)	0.099
**Actinobacteria**	0.0345 (0.0173 - 0.0612)	0.0185 (0.0156 - 0.0483)	0.187
Bifidobacterium adolescentis	0.0035 (0.0003 - 0.0198)	0.0004 (0.0000 - 0.0112)	0.174
Bifidobacterium kashiwanohense	0.0000 (0.0000 - 0.0011)	0.0000 (0.0000 - 0.0003)	0.214
Bifidobacterium longum	0.0051 (0.0017 - 0.0106)	0.0050 (0.0008 - 0.0107)	0.850
Bifidobacterium merycicum	0.0000 (0.0000 - 0.0000)	0.0000 (0.0000 - 0.0000)	0.851
Bifidobacterium pseudocatenulatum	0.0005 (0.0000 - 0.0038)	0.0000 (0.0000 - 0.0013)	0.147
**Verrucomicrobia**	0.0004 (0.0000 - 0.0034)	0.0004 (0.0000 - 0.0043)	0.698
Akkermansia muciniphila	0.0004 (0.0000 - 0.0049)	0.0004 (0.0000 - 0.0043)	0.554
**Proteobacteria**	0.0128 (0.0057 - 0.0196)	0.0123 (0.0065 - 0.0236)	0.812
**Synergistetes**	0.0000 (0.0000 - 0.0000)	0.0000 (0.0000 - 0.0000)	0.810
**Fusobacteria**	0.0000 (0.0000 - 0.0000)	0.0000 (0.0000 - 0.0000)	0.350
**Bacteria "**unspecified phylum"	0.0003 (0.0000 - 0.0009)	0.0000 (0.0000 - 0.0006)	0.521
**F/B** (ratio)	0.3016 (0.2037 - 0.4681)	0.2515 (0.2130 - 0.3678)	0.421

Note: Data presented as mean ± SD for normal continuous variables, median (1st quartile – 3rd quartile) for non-normal continuous variables; *p*-value <0.05 considered significant. Bacteria written in bold indicate phylum while in italic indicate species. Firmicutes/Bacteroidetes (F/B).

Supplementary Table S2. Lifestyle Parameters of Participants According to Body Mass Index and Vitamin D status[1]

Variables	Non-Obese			Obese		
	**Vitamin D >25 nmol/l**	**Vitamin D < 25 nmol/l**	***P*-value**	**Vitamin D >25 nmol/l**	**Vitamin D < 25 nmol/l**	***P*-value**
**Dietary Intake**						
Energy (kcal/day)	3613.2 (2709.9 - 4404.8)	4649.3 (3358.1 - 5262.9)	0.095	2805.2 (2240.1 - 4215.2)	3506.0 (2856.3 - 4261.2)	0.235
Fat intake (%of total kcal)	36.6 (32.7 - 40.2)	45.7 (36.2 - 52.2)	**0.029**	41.6 (33.1 - 44.6)	36.6 (30.1 - 46.7)	0.720
Protein intake (%of total kcal)	14.4 (12.8 - 16.2)	11.8 (10.0 - 13.3)	**0.004**	14.6 (12.0 - 17.3)	12.1 (11.3 - 13.5)	**0.034**
Carbohydrate intake (%of total kcal)	47.7 (40.6 - 53.1)	41.7 (37.9 - 51.4)	0.179	42.3 (40.8 - 48.8)	51.8 (41.1 - 57.6)	0.185
Fiber (g/1000kcal)	10.2 (7.9 - 12.4)	9.4 (9.1 - 10.8)	0.455	10.0 (8.5 - 11.3)	8.3 (7.1 - 10.7)	0.092
Vitamin D (mcg/day)	3.6 (2.8 - 5.3)	4.8 (2.0 - 6.1)	0.492	4.2 (2.2 - 6.7)	4.0 (2.6 - 6.2)	0.720
Potassium (mg/1000kcal)	1908.6 (1633.0 - 2774.0)	1881.7 (1312.1 - 2119.1)	0.232	1925.0 (1488.9 - 3252.5)	1395.3 (959.7 - 1846.7)	**0.025**
Calcium (mg/1000kcal)	48.1 (9.9 - 91.6)	25.3 (9.0 - 42.4)	0.387	86.4 (44.3 - 173.3)	52.2 (3.5 - 86.3)	**0.022**
Phosphors (mg/1000kcal)	663.2 (575.7 - 941.7)	587.3 (506.9 - 677.8)	0.107	628.3 (535.2 - 907.1)	534.9 (403.6 - 634.2)	**0.034**
Iron (mg/1000kcal)	14.3 (10.2 - 20.8)	11.2 (9.8 - 14.0)	0.210	11.6 (9.4 - 22.0)	9.9 (7.0 - 14.6)	0.167
**Physical Activity Parameters**						
Total activity energy expenditure (METs-min/wk)	720.0 (360.0 - 1280.0)	400.0 (120.0 - 708.0)	0.078	570.0 (344.0 - 1780.0)	402.0 (210.0 - 1080.0)	0.306
Sitting time(min/day)	465.0 (300.0 - 600.0)	300.0 (240.0 - 420.0)	0.260	420.0 (300.0 - 570.0)	390.0 (300.0 - 600.0)	0.627
**Sleep**						
Overall PSQI Score						
Good	14 (41.2)	1 (11.1)	0.129	4 (16.7)	1 (6.3)	0.631
Poor	20 (58.8)	8 (88.9)		20 (83.3)	15 (93.8)	
**Sun Exposure**						
**Sun Exposure Minute Per Day (mean±SD)**	9.3 (5.0 - 15.0)	10.0 (4.0 - 15.0)	0.399	10.0 (5.0 - 17.5)	12.5 (6.0 - 20.0)	0.469
**Noon time sun exposure**						
Yes	4 (11.8)	4 (44.4)	**0.046**	11 (45.8)	4 (25.0)	
No	30 (88.2)	5 (55.6)		13 (54.2)	12 (75.0)	0.182

[1]Data presented as mean ± SD for normal variables whereas median (1st quartile – 3rd quartile) for non-normal variables; # indicates non-normal variables; p<0.05 considered significant. Gram (gm), Milligram (mg), and Microgram (mcg).

Supplementary Table S3. Correlation coefficients between vitamin D intake and gut microbiota among study groups

Variables		Vitamin D intake	R	*p*-value*
**Gut Microbiota**	**Total participants**	*Bifidobacterium kashiwanohense*	-0.22	0.04
	**Normal group**	*Blautia wexlerae*	-0.21	0.05
		Akkermansia muciniphila	0.29	0.03
		Flavonifractor plautii	-0.33	0.02
	**Obese group**	*Bacteroides* "unspecified species"	0.35	0.02
		Blautia wexlerae	-0.30	0.05
		Akkermansia muciniphila	-0.32	0.04
		Actinobacteria phylum	-0.35	0.02

*** *p*-value < 0.05 considered significant.**

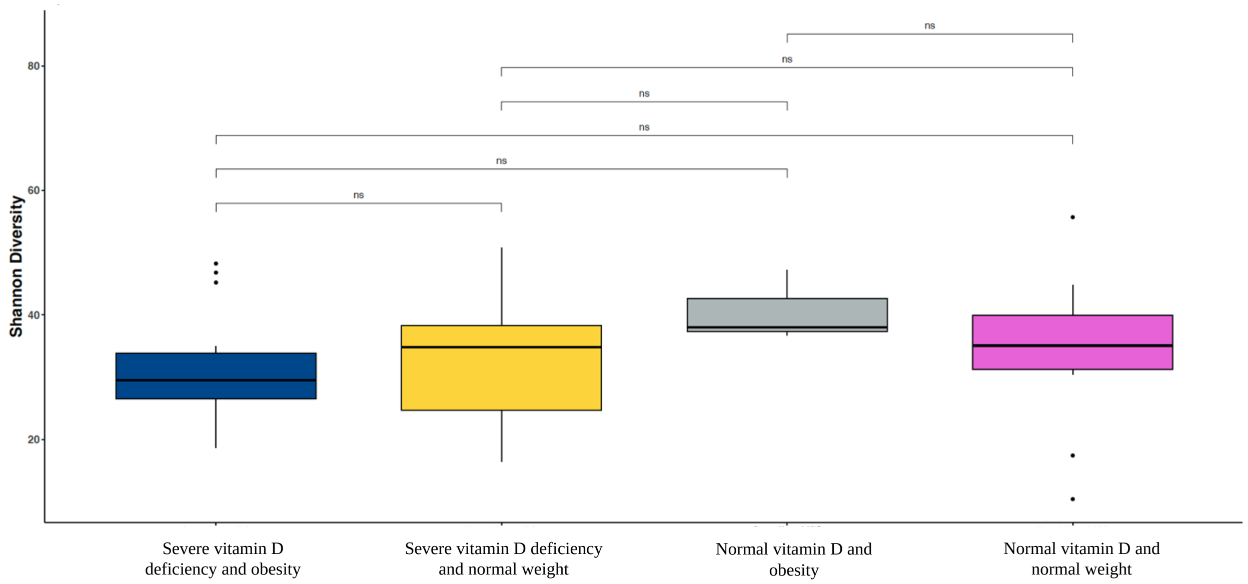

Supplementary Figure S1. Comparison of bacterial diversity (Shannon-Wiener index) between the microbiota at the species level of group with obesity and group with normal weight based on BMI and severe vitamin D deficiency (< 25 nmol/l).

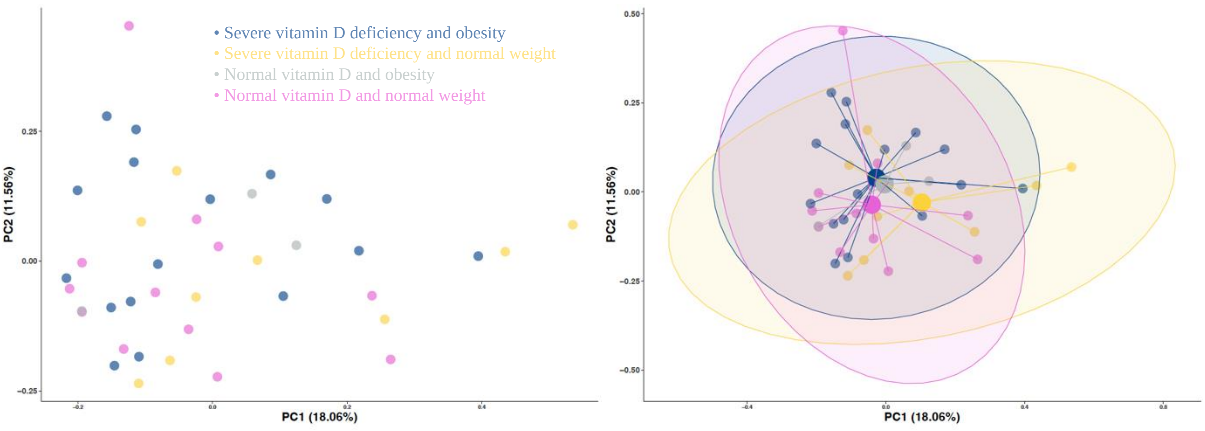

Supplementary Figure S2. Comparison of bacterial diversity (PERMANOVA Analysis) between the microbiota at the species level of normal weight and participants with obesity based on BMI and severe vitamin D deficiency (< 25 nmol/l).
